# Changes in Air Quality and Drivers for the Heavy PM_2.5_ Pollution on the North China Plain Pre- to Post-COVID-19

**DOI:** 10.3390/ijerph191912904

**Published:** 2022-10-08

**Authors:** Shuang Liu, Xingchuan Yang, Fuzhou Duan, Wenji Zhao

**Affiliations:** College of Resource Environment and Tourism, Capital Normal University, Beijing 100048, China

**Keywords:** air quality, PM_2.5_, gaseous pollutants, meteorological factors, North China Plain, COVID-19

## Abstract

Under the clean air action plans and the lockdown to constrain the coronavirus disease 2019 (COVID-19), the air quality improved significantly. However, fine particulate matter (PM_2.5_) pollution still occurred on the North China Plain (NCP). This study analyzed the variations of PM_2.5_, nitrogen dioxide (NO_2_), sulfur dioxide (SO_2_), carbon monoxide (CO), and ozone (O_3_) during 2017–2021 on the northern (Beijing) and southern (Henan) edges of the NCP. Furthermore, the drivers for the PM_2.5_ pollution episodes pre- to post-COVID-19 in Beijing and Henan were explored by combining air pollutant and meteorological datasets and the weighted potential source contribution function. Results showed air quality generally improved during 2017–2021, except for a slight rebound (3.6%) in NO_2_ concentration in 2021 in Beijing. Notably, the O_3_ concentration began to decrease significantly in 2020. The COVID-19 lockdown resulted in a sharp drop in the concentrations of PM_2.5_, NO_2_, SO_2_, and CO in February of 2020, but PM_2.5_ and CO in Beijing exhibited a delayed decrease in March. For Beijing, the PM_2.5_ pollution was driven by the initial regional transport and later secondary formation under adverse meteorology. For Henan, the PM_2.5_ pollution was driven by the primary emissions under the persistent high humidity and stable atmospheric conditions, superimposing small-scale regional transport. Low wind speed, shallow boundary layer, and high humidity are major drivers of heavy PM_2.5_ pollution. These results provide an important reference for setting mitigation measures not only for the NCP but for the entire world.

## 1. Introduction

Air pollution over the past decade has been an increasing concern for the public and central government, especially concerning persistent and severe fine particulate matter (PM_2.5_) pollution [1,2,3]. Extensive epidemiological studies have shown that long-term exposure to air pollution can increase the incidence of respiratory and cardiovascular diseases [4,5,6]; therefore, prevention and control measures to mitigate air pollution are urgently required. As a response, the State Council promulgated the “Atmospheric Pollution Prevention and Control Action Plan” in September 2013, which targeted reducing PM_2.5_ pollution during 2013–2017. Subsequently, the State Council issued the “Three-year Action Plan on Defending the Blue Sky”, which further focused on greater achievements in alleviating air pollution during 2018–2020. Specifically, the North China Plain (NCP) is the most polluted region in China, and tremendous pollution controls have been implemented to achieve the targets set by the State Council on the NCP. Major control measures included shutting down small and polluting factories, strengthening industrial and vehicle emission standards, upgrading industrial boilers, and promoting clean energy [7,8]. As air pollution is a regional issue, a regional joint mitigation measure called “2 + 26” strategies, which comprised two megacities (Beijing and Tianjin) and 26 neighboring cities on the NCP [9], was promulgated in September 2017. After the two stages of clean air action plans, air pollution has improved significantly [10,11,12,13,14].

Unexpectedly, Wuhan City, the capital of Hubei Province in China, reported the appearance of coronavirus disease 2019 (COVID-19) in late December 2019 [15,16]. COVID-19 spread rapidly to the rest of China and other countries in the ensuing months and has become a global health issue until now. Wuhan City first implemented all lockdown measures on 23 January 2020, and subsequently, lockdown measures were implemented across China, including wearing masks, social distancing, travel bans, home quarantine, and national lockdowns. Despite the drawbacks of COVID-19, it had a positive impact on air quality due to the reduction in anthropogenic emissions [17,18]. Worldwide, extensive studies compared air quality before and during the COVID-19 lockdown [19,20,21,22,23,24,25,26]. Interestingly, there was a marked decline in particulate matter with an aerodynamic diameter of less than 10 μm (PM_10_), PM_2.5_, sulfur dioxide (SO_2_), nitrogen dioxide (NO_2_), and carbon monoxide (CO) during the worldwide COVID-19 lockdown [26,27,28,29]. The NO_2_ concentration reduced by about 20% in China over 30–50 days [30]. Bao and Zhang et al. [31] found that the average air quality index (AQI) decreased by 7.8%, and the concentrations of PM_10_, PM_2.5_, SO_2_, NO_2_, and CO decreased by 13.7%, 5.9%, 6.8%, 24.7%, and 4.6%, respectively, for 44 cities in northern China. Bhatti et al. [32] further assessed the changes in ambient air quality in Jiangsu of China pre- to post-COVID-19. By contrast, studies around the world reported an uneven behavior in ozone (O_3_) concentration during lockdown [33]. Most studies reported an increase in O_3_ concentration [34,35,36,37], while several studies revealed a decrease [38,39,40]. Moreover, several studies found that O_3_ has no significant variations in some cities in Spain, the kingdom of Saudi Arabia, Italy, and Greece [33,41,42,43]. Although COVID-19 affects humans severely, the pandemic has been effectively controlled by vaccines and other control measures in China. Everything is gradually returning to normal. Consequently, a comprehensive understanding of the variations in air quality pre- to post-COVID is important for policymakers. However, studies on the longer-term variations in air pollutants are limited, especially in key regions.

Air pollution on the NCP has been a research hotspot due to frequent haze pollution, characterized by the accumulation of PM_2.5_. The formation of PM_2.5_ results from direct emission from primary sources and efficient secondary transformation of aerosols from precursor gases [44,45,46,47,48,49]. The main gaseous pollutants include NO_2_, SO_2_, CO, and O_3_ [50]. NO_2_ and SO_2_ act as gaseous precursors to form PM_2.5_ through heterogeneous atmospheric nucleation [14]. NO_2_ is mainly from on-road vehicle emissions [51], and the major sources of SO_2_ are power plants, industrial emissions, and domestic heating [52]. CO is the primary pollutant emitted by combustion processes. Particularly, surface ozone is a secondary air pollutant and is produced by the photochemical reactions of nitrogen oxide precursors (NO_x_) and volatile organic compounds (VOCs) in the presence of sunlight [50,53]. Previous studies have revealed the emission sources of PM_2.5_ on the NCP [54,55,56,57,58], involving diverse pollution sources from vehicle emissions, industrial production, construction and road dust emissions, biomass burning, fossil fuel combustion, and the use of chemical pesticides and solvents. In particular, vehicle exhaust, industrial production, and fossil fuel combustion were the dominant sources of PM_2.5_ [59,60].

Understanding the drivers for heavy PM_2.5_ pollution is essential while making policies to mitigate PM_2.5_ pollution. Generally, PM_2.5_ pollution is associated with enormous emissions [61], unfavorable meteorology [62,63], enhanced secondary formation [64,65], and regional transport [66,67]. Meteorological drivers play important roles in air pollution formation, deposition, transport, and transformation [62,63]. Existing studies revealed meteorological causes for the deteriorated PM_2.5_ pollution [68,69,70], including weak surface winds, shallow planetary boundary layer, and high humidity that favored the accumulation and secondary formation of PM_2.5_. The adverse meteorology could contribute to PM_2.5_ pollution even if the emissions decreased. Notably, heavy PM_2.5_ pollution episodes were observed in January and February of 2020 and 2021 over the NCP, during which anthropogenic emissions decreased significantly due to the outbreak of COVID-19 and the Spring Festival [13,66,71]. Related studies revealed that the PM_2.5_ pollution episodes during this special period were attributed to enhanced secondary formation under a stagnant atmosphere [13,65,70,72]. These studies mainly focused on Beijing and its surrounding areas on the northern edge of the NCP. Henan is located on the southern edge of the NCP and has also been suffering from heavy PM_2.5_ pollution. Moreover, seven cities within Henan are located in the Beijing-Tianjin-Hebei (BTH) transmission corridor. However, only a few studies investigated the variations and formation mechanisms of PM_2.5_ pollution in Henan. Liu et al. [58] revealed that severe haze events were tightly related to adverse meteorological, atmospheric transformation processes, and regional transport. Song et al. [73] indicated that PM_2.5_ deterioration during PM_2.5_ pollution episodes was associated with local emissions and secondary formation on the south edge of the NCP, which was different from the northern edge. Moreover, studies on heavy PM_2.5_ pollution episodes during active- to post-COVID-19 remain lacking.

To fill the abovementioned gaps, this study aims to comprehensively assess the changes in concentrations of PM_2.5_ and gaseous pollutants from 2017 to 2021 based on the observation data over the NCP, focusing on Beijing on the northern edge of the NCP and Henan on the southern edge of the NCP. Further, the drivers for the heavy PM_2.5_ pollution episodes were explored in Beijing and Henan pre- to post-COVID-19. Specifically, the three main objectives of the study were to: (1) analyze the variation trends of PM_2.5_ and gaseous pollutants during 2017–2021 in Beijing and Henan; (2) determine the PM_2.5_ pollution status when anthropogenic emissions significantly decreased, especially between 2019 and 2021; (3) explore the formation mechanism of heavy PM_2.5_ pollution on the northern and southern edges of the NCP between 2019–2021. The findings gained from the present study would provide an important theoretical reference for future air pollution prevention strategies over the NCP.

## 2. Materials and Methods

### 2.1. Study Region

Beijing, as the capital of China, is the political and cultural center and is located on the northern edge of the NCP. With the dramatic economic development, Beijing has become one of the well-developed megacities. Along with economic development, the ensuing problem of air pollution was not ignored. Notably, the high emissions plants were relocated out of Beijing, and cleaner production techniques were employed to alleviate industrial emissions [52]. Luo et al. [74] indicated that vehicular and secondary sources have become much more dominant recently. In contrast, Henan is located on the southern edge of the NCP and is a developing province. Henan is one of the largest energy-consuming provinces in China, with coal as its primary energy source [54]; hence, Henan faces greater challenges in balancing development and the ecological environment. Industrial, transportation, and energy consumption contribute to substantial PM_2.5_ emissions [75]. Moreover, Henan is the second largest agricultural province in China, accounting for 25% of China’s wheat output every year [76], and planting and agriculture are important emission sources of air pollution.

### 2.2. Ambient Air Pollutants and Meteorological Dataset

The ground-level monitoring network of ambient air pollutants established by the Ministry of Ecology and Environment of China began operating in 2013. In the present study, ground-level hourly concentrations of five ambient air pollutants in Beijing and Henan were employed, including PM_2.5_, NO_2_, SO_2_, CO, and O_3_. PM_2.5_ was measured via the micro-oscillating balance method. For gaseous pollutants, the NO_2_, SO_2_, CO, and O_3_ were monitored using chemiluminescence, ultraviolet fluorescence, non-dispersive infrared absorption, and ultraviolet spectrometry, respectively. The ambient air pollutant data was released by the China National Environmental Monitoring Centre, and was collected from the online database (https://quotsoft.net/air/#archive, accessed on 27 July 2022). The daily-average concentrations of PM_2.5_, NO_2_, SO_2_, and CO were computed as the 24-h mean values for each site. As for O_3_, the maximum daily 8-h average (MDA8) concentration was calculated. Moreover, the monthly and yearly concentrations were acquired based on the daily levels.

**Figure 1:**
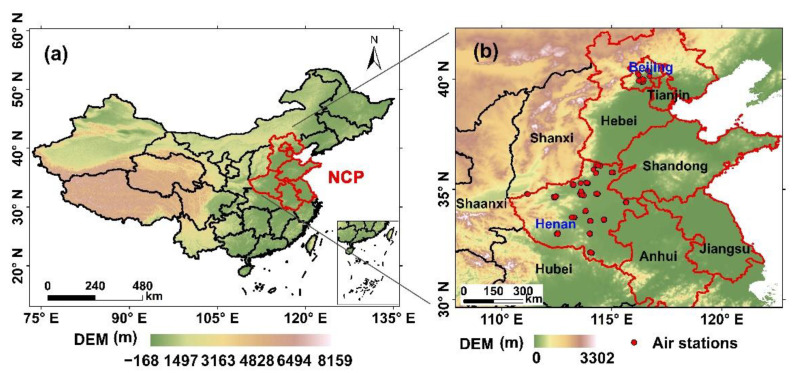
(**a**) Map of study area, and (**b**) locations of the air-quality monitoring sites in Beijing and Henan.

The present study aims to highlight the variation trends of PM_2.5_ and gaseous pollutants and the drivers for the persistent PM_2.5_ pollution episodes during pre- to post-COVID (2019–2021). Simultaneously, the pollution levels during 2017–2018 were analyzed to evaluate the variations in air pollutants comprehensively. Considering the continuous development of air pollutant monitoring stations, the monitoring sites that remained the same between 2019 and 2021 were selected in this study, as shown in Figure 1, with 12 sites in Beijing and 83 sites in Henan, respectively. Table S1 provides details of the site locations. Meanwhile, for 2017 and 2018, the sites that remained the same between the two years were chosen, with 12 sites in Beijing and 75 sites in Henan, respectively. Sites were selected this way to obtain as much data as possible while ensuring data consistency. These site types involve urban, industrial, residential, traffic, educational, and park sites, which could provide a comprehensive assessment of air quality in the study area.

The meteorological data include boundary layer height (BLH), 10 m wind components u and v, temperature and dewpoint temperature at 2 m during 2019–2021, and were obtained from the European Center for Medium-Range Weather Forecasts (ECMWF) ERA5 hourly reanalysis dataset (http://cds.climate.copernicus.eu/cdsapp#/home, accessed on 27 July 2022), with a resolution of 0.25° × 0.25°. Further, relative humidity (RH) was calculated employing temperature and dewpoint temperature at 2 m. Additionally, meteorological inputs to the backward trajectories were 1° × 1° Global Data Assimilation System (GDAS) data from the National Centers for Environmental Prediction (NCEP) reanalysis (available at ftp://arlftp.arlhq.noaa.gov/pub/archives/gdas1, accessed on 27 July 2022).

### 2.3. Potential Sources Analysis

To investigate the origin of airborne particles during heavy PM_2.5_ pollution episodes, 24-h backward trajectories were calculated four times each day (00:00, 06:00, 12:00, and 18:00 UTC) at the height of 500 m above the ground in Beijing (39.87° N, 116.43° E) and Henan (34.75° N, 113.63° E), based on the Hybrid Single Particle Lagrangian Integrated Trajectory (HYSPLIT) model developed by the National Oceanic and Atmospheric Administration (NOAA) Air Resources Laboratory (ARL) [77]. Based on the backward trajectories, the potential source contribution function (PSCF) method was applied to evaluate the potential geographic origins for PM_2.5_ pollution episodes in Beijing and Henan [78]. Prior to conducting the PSCF analysis, the geographic region covered by the backward trajectories was divided into an array of 0.5° × 0.5° grid cells. The PSCF is a conditional probability function [66]. Specifically, the PSCF value in the grid (*i*, *j*) is calculated as *M_ij_*/*N_ij_*, where *i* and *j* are the latitude and longitude, *N_ij_* is the number of endpoints that fall in the grid (*i*, *j*), and *M_ij_* is the number of polluted trajectory endpoints in the grid (*i*, *j*). The daily PM_2.5_ concentration exceeding 75 μg m^−3^ refers to a polluted day according to the new China National Ambient Air Quality Standards issued in 2012. In the present study, the polluted trajectory was thus determined to exceed a threshold concentration of 75 μg m^−3^. High PSCF values represent higher probabilities of grid cells being potential geographic origins, making a greater contribution to air pollution within a receptor region. When calculating the PSCF values, Zeng and Hopke [79] found some grid cells only had one endpoint (*N_ij_* = 1). If this endpoint corresponds to a polluted trajectory, the PSCF values for these grid cells will be 1. However, the confidence in these PSCF values is very low. Therefore, to reduce the PSCF uncertainties caused by the small values of *N_ij_*, an arbitrary weight factor (*W_ij_*) was multiplied by the PSCF value [80]. The weighted PSCF (WPSCF) was calculated as *WPSCF_ij_* = *PSCF_ij_* × *W_ij_*. The *W_ij_* is defined as follows [81,82],

(1) $$W_{ij}=\{\begin{array}{c} 1.00\;(80<N_{ij})\\ 0.72\;(20<N_{ij}\;\leq 80)\\ 0.42\;(10<N_{ij}\leq 20)\\ 0.05\;\left(N_{ij}\;\leq 10\right) \end{array}.$$

## 3. Results and Discussions

### 3.1. Variation Trends of PM_2.5_ and Gaseous Pollutants

#### 3.1.1. Yearly Variations

**Figure 2:**
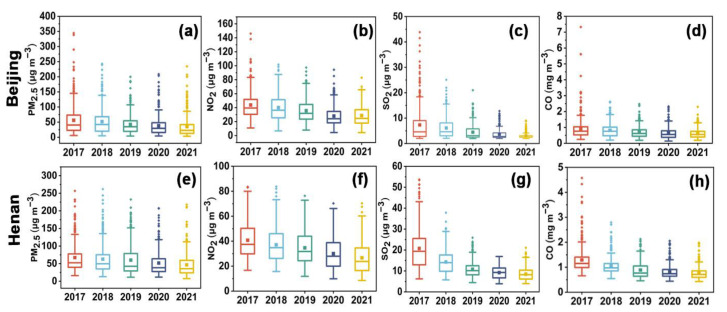
Yearly variations in the concentrations of PM_2.5_, NO_2_, SO_2_, and CO during 2017–2021 in Beijing and Henan. (**a**–**d**) show annual concentrations of PM_2.5_, NO_2_, SO_2_, and CO during 2017–2021 in Beijing, respectively, and (**e**–**h**) show annual concentrations of PM_2.5_, NO_2_, SO_2_, and CO during 2017–2021 in Henan, respectively.The box frames represent the upper and lower quartile, the line represents the median, the whiskers denote the range within 1.5IQR, the square point within the box frame represents the mean, and the points outside the box frame represent outliers.

Figure 2 presents the annual variations of PM_2.5_ and gaseous pollutants (NO_2_, SO_2_, CO, and O_3_) between 2017 and 2021 in Beijing and Henan, along with their annual descriptive statistics in Table S2. The annual concentrations of PM_2.5_ decreased dramatically in Beijing and Henan from 2017 to 2021. Specifically, compared to the average concentrations of PM_2.5_ in Beijing (56 μg m^−3^) and Henan (67 μg m^−3^) in 2017, their concentrations in 2021 decreased to 35 μg m^−3^ and 47 μg m^−3^, respectively. For 2018–2021, the PM_2.5_ concentrations in Beijing decreased by 8.9%, 17.6%, 9.5%, and 7.9%, respectively, compared to the previous year. Similarly, the PM_2.5_ concentrations in Henan decreased by 6.0%, 4.8%, 13.3%, 9.6%, respectively. For Beijing, the decline rate (17.6%) from 2018 to 2019 was the highest. However, the highest decline rate (−13.3%) in Henan was observed from 2019 to 2020, with a one-year delay compared to Beijing. Furthermore, it is noted that the average PM_2.5_ concentrations in Henan were significantly higher than that in Beijing.

As for NO_2_, the annual-average concentrations in Beijing showed a decreasing trend during 2017–2020, from 44 μg m^−3^ in 2017 down to 28 μg m^−3^ in 2020. Particularly, the reduction in 2020 was the largest, with a decrease rate of 22.2%. However, the annual average concentrations of NO_2_ rebounded slightly in 2021, increasing by 3.6% relative to 2020. Unlike Beijing, the annual concentrations of NO_2_ in Henan exhibited a continuous decreasing trend during the study period, from 41 μg m^−3^ in 2017 to 27 μg m^−3^ in 2021. The most pronounced reduction occurred in 2020 (−14.3%), followed by 2021 (−10.0%). In contrast to PM_2.5_, NO_2_ pollution levels were higher in Beijing than in Henan, except in 2020.

For SO_2_ and CO, the annual levels in Beijing and Henan exhibited descending trends during 2017–2021. The annual concentrations of SO_2_ in Beijing decreased from 7 μg m^−3^ in 2017 to 3 μg m^−3^ in 2021, and the annual concentrations of CO decreased from 0.9 mg m^−3^ in 2017 to 0.6 mg m^−3^ in 2021. In particular, the most obvious decline in annual concentrations was observed in 2019 for SO_2_ (−33.3%) and in 2020 for CO (−14.3%). Compared with Beijing, the annual concentrations of SO_2_ and CO were higher in Henan. The average SO_2_ concentration in Henan decreased from 21 μg m^−3^ in 2017 to 8 μg m^−3^ in 2021, and the average CO concentration decreased from 1.3 mg m^−3^ in 2017 to 0.7 mg m^−3^ in 2021. Significantly, the larger decline occurred in 2018 for SO_2_ (−33.3%) and in 2019 for CO (−25.0%).

Furthermore, the variations of O_3_ during the 2017–2021 period were analyzed. In Beijing, the 90th percentiles of MDA8 O_3_ values fluctuated slightly from 2017 to 2019, then decreased by 10.2% to 176 μg m^−3^ in 2020, and further decreased by 10.2% to 158 μg m^−3^ in 2021. Like Beijing, the 90th percentiles of MDA8 O_3_ values in Henan fluctuated slightly during the first three years of the study period. Subsequently, the 90th percentiles of MDA8 O_3_ values decreased to 168 μg m^−3^ in 2020 and reduced further in 2021 to 161 μg m^−3^.

#### 3.1.2. Monthly Variations

**Figure 3:**
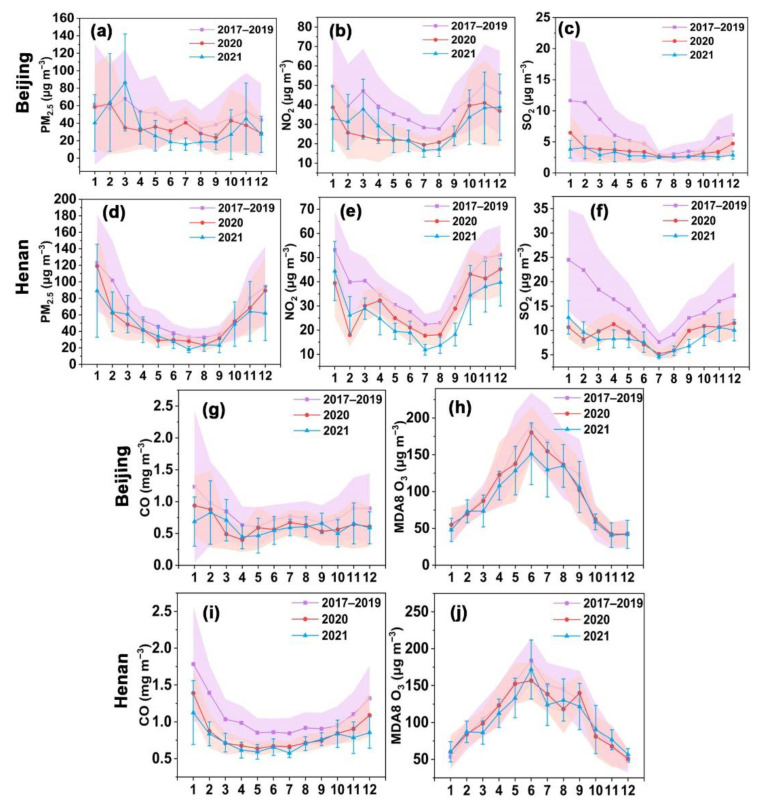
Monthly variations in the concentrations of PM_2.5_, NO_2_, SO_2_, CO, and MDA8 O_3_ during 2017–2021 in Beijing and Henan. (**a**–**c**,**g**,**h**) show monthly concentrations of PM_2.5_, NO_2_, SO_2_, CO, and MDA8 O_3_ during 2017–2021 in Beijing, respectively, and (**d**–**f**,**i**,**j**) show monthly concentrations of PM_2.5_, NO_2_, SO_2_, CO, and MDA8 O_3_ during 2017–2021 in Henan, respectively. The light-shaded areas or error bars represent the standard deviation.

The monthly variations of PM_2.5_ and gaseous pollutants (NO_2_, SO_2_, CO, and O_3_) in Beijing and Henan were further analyzed (Figure 3). Notably, the monthly average concentrations of air pollutants during 2017–2019 were calculated as the historical levels before COVID-19, and the present study highlighted the monthly variations of air pollutants during and post-COVID-19 (2020–2021). The monthly variations of PM_2.5_ concentrations exhibited a U-shaped pattern in Henan, which was consistent with the results of Yao et al. [54]. Unlike Henan, the monthly average concentrations of PM_2.5_ in Beijing fluctuated, and differences between winter and summer were relatively small, without showing a pronounced U-shaped pattern. This may be attributed to a series of strict air pollution control measures in Beijing during recent years, which has dramatically improved PM_2.5_ pollution, especially in winter with higher PM_2.5_ concentrations.

In 2020, the average PM_2.5_ concentration in Beijing increased slightly by 6.91% to 62.47 μg m^−3^ in February relative to January. Subsequently, the PM_2.5_ concentration reduced sharply (−44.35%) in March compared to February and remained relatively stable before increasing in October. Gao et al. [83] also revealed a similar phenomenon; that is, PM_2.5_ concentration began to decline in March of 2020. Moreover, the monthly PM_2.5_ concentrations showed a decreasing trend in November and December. Notably, the monthly average concentration in December was relatively low, at 28.51 μg m^−3^. The monthly concentrations in 2021 were generally at a lower level compared to 2017–2020, except for March. It is worth noting that PM_2.5_ concentration in March of 2020 was significantly lower than in historical years (2017–2019) and 2021. However, the PM_2.5_ concentration in March of 2021 reached a peak (86.59 μg m^−3^). In Henan, the PM_2.5_ concentration in February dropped significantly by 47.54% relative to January 2020. Compared with the corresponding monthly averages in 2020, the monthly averages in 2021 in Henan generally showed minor fluctuations.

As for gaseous pollutants, the monthly-average concentrations of NO_2_, SO_2_, and CO were generally higher from October to March than those from April to September, while O_3_ showed an opposite trend due to its formation mechanism. The increase in temperature and solar radiation favored the photochemical formation of O_3_ from VOCs and NO_x_ between April and September [84,85]. Meanwhile, the reduction in NO_x_ levels contributed to higher O_3_ levels by hindering the NO_x_ titration and/or the effect of radical terminating reactions [19]. It is worth noting that the NO_2_ concentrations in February decreased generally compared with that in January. Especially in 2020, the monthly-average concentration of NO_2_ in February dropped sharply by 33.58% in Beijing and subsequently remained stable, while that in August started to increase significantly. In contrast with 2020, the monthly NO_2_ concentration in March during the period of historical years (2017–2019) and 2021 rebounded rapidly. Notably, the monthly NO_2_ concentration between February and May in 2020 was the lowest compared to 2017–2019 and 2021. Similar to Beijing, NO_2_ concentration in Henan decreased sharply by 54.49% in February 2020 relative to January. However, NO_2_ concentration rebounded rapidly in March, increasing by 67.06%. The monthly averages of NO_2_ in 2021 generally decreased relative to 2017–2020, while the averages in January and February of 2021 were higher than in 2020, especially in February.

Generally, the NO_2_ concentration is lower in February relative to January, as many household heating and industrial activities decline around this time of year due to the Spring Festival [70]. However, the sharper drop in NO_2_ concentration during February 2020 in Beijing and Henan was mainly attributed to lockdown measures to constrain COVID-19 [70]. Furthermore, a study has revealed that air pollution in Beijing and its neighboring provinces recovered more slowly as a consequence of the extension of the lockdown [86]. This also validates our finding that NO_2_ concentrations in Beijing rebounded more slowly.

With regard to SO_2_, the variation trend of monthly average concentration in 2020 was generally similar to that of NO_2_ in Beijing and Henan. Overall, the monthly average concentration of SO_2_ in 2021 showed a downward trend compared to 2020 in Beijing. For Henan, the monthly average concentration deteriorated in January and February of 2021 relative to 2020; the monthly concentration between March and December in 2021 presented a decreasing trend relative to the corresponding months in 2020. As for CO, the monthly concentration in February 2020 decreased dramatically by 37.14% in Henan. Unlike Henan, the sharp decrease in CO in Beijing occurred in March 2020, with a drop of 44.24%, reaching the lowest value (0.49 mg m^−3^) compared with 2017–2019 and 2021. The variations in CO in Beijing and Henan were consistent with PM_2.5_, and Liu et al. [14] have revealed a strong correlation between PM_2.5_ and CO. The delayed sharp decrease in Beijing was attributed to the transported pollutants emitted by non-stop industrial emissions and fireworks, as well as the effect of unfavorable meteorological conditions [83].

### 3.2. Analysis of PM_2.5_ Pollution Conditions

The changes in PM_2.5_ pollution conditions were investigated in Beijing and Henan during the period of 2017–2021. According to the China National Ambient Air Quality Index (AQI) Technical Regulations, the daily mean PM_2.5_ concentration level can be divided into excellent (≤35 μg m^−3^), good (35–75 μg m^−3^), light pollution (75–115 μg m^−3^), moderate pollution (115–150 μg m^−3^), heavy pollution (150–250 μg m^−3^), and severe pollution (>250 μg m^−3^). Based on this, the proportion of days with different concentration levels was calculated from 2017 to 2021 in Beijing and Henan (Table 1). In Beijing, the days with severe pollution accounted for 1.12% in 2017. It is worth noting that there have been no severe pollution days in Beijing since 2018. Moreover, the percentage of days with excellent levels increased significantly, with 50.3% (2019), 57.8% (2020), and 69.4% (2021) in the last three years, respectively. However, heavy pollution episodes still occurred during 2019–2021, although their proportion was significantly lower than in 2017 (4.5%) and 2018 (4.2%). In Henan, severe pollution episodes have been eliminated since 2019. The proportion of days with excellent levels increased significantly, while the proportion of good levels showed a decreasing trend. The days with good levels gradually changed to excellent days. Like Beijing, Henan persistently suffered from heavy PM_2.5_ pollution from 2017 to 2021, with a lower proportion in 2020 (2.2%) and 2021 (1.9%).

In comparison to gaseous precursors, the composition of PM_2.5_ is more complicated. As mentioned above, PM_2.5_ pollution has been mitigated under a set of policies and control measures in recent years, while haze still occurred on the NCP. Notably, PM_2.5_ pollution was observed in January and February of 2020, when anthropogenic emissions decreased significantly due to the outbreak of COVID-19 and the Spring Festival [66,71]. Therefore, this study next focused on the period of pre- to post-COVID-19 (2019–2021) and analyzed the drivers for the PM_2.5_ pollution episodes on the northern and southern edges of the NCP in the past three years.

### 3.3. Analysis of PM_2.5_ Pollution Episodes Pre-, during and Post-COVID

#### 3.3.1. Analysis of PM_2.5_ Pollution Episodes on the Northern Edge of the NCP

**Figure 4:**
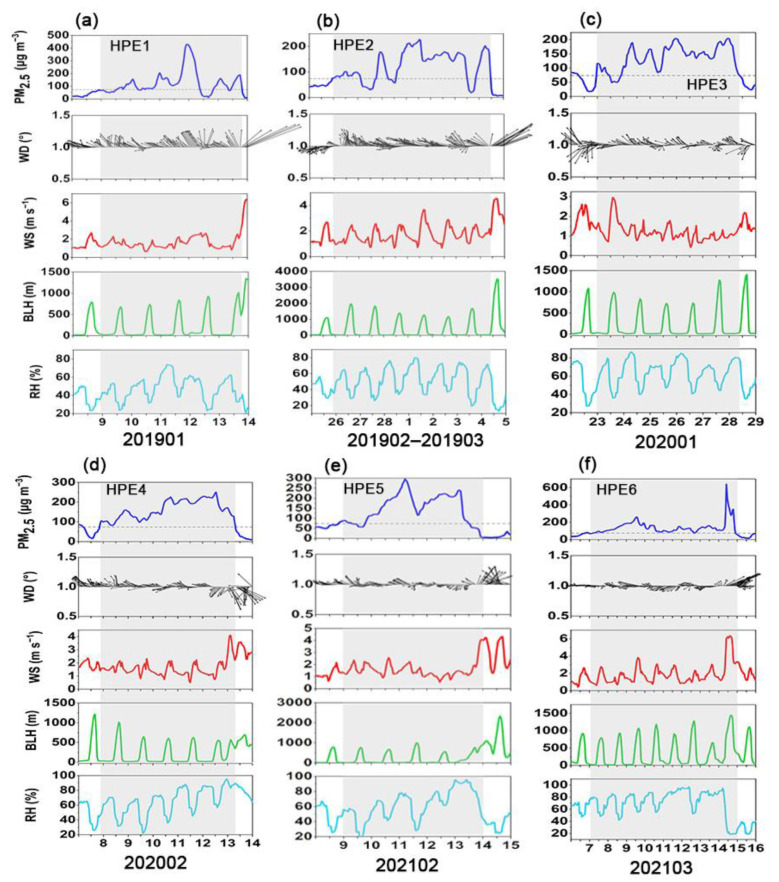
Time series of PM_2.5_ concentrations, wind direction (WD), wind speed (WS), boundary layer height (BLH), and relative humidity (RH) during six heavy PM_2.5_ pollution episodes (HPEs) in Beijing. (**a**) HPE1, (**b**) HPE2, (**c**) HPE3, (**d**) HPE4, (**e**) HPE5, and (**f**) HPE6.

The potential causes were explored for six heavy PM_2.5_ pollution episodes (HPEs) in Beijing between pre-COVID in 2019 and post-COVID in 2021 (Table 2). Notably, HPE6 lasted for eight days, followed by HPE2 (seven days), and the remaining pollution incidents lasted for five days. The evolution of PM_2.5_ concentration, wind direction (WD), wind speed (WS), BLH, and RH during six pollution episodes is shown in Figure 4. Generally, the HPEs were initially caused by southerly or southeasterly winds, which transported air pollutants from southern regions to Beijing [87,88,89]. Hence, PM_2.5_ began accumulating in Beijing, and PM_2.5_ concentration gradually increased. Subsequently, the BLH dropped significantly compared to the clean period, and the RH increased simultaneously, above 60%. Notably, the highest RH exceeded 80%. The WS was low, and the average WS was less than 2 m s^−1^. Therefore, PM_2.5_ pollution further deteriorated due to the stagnant meteorological conditions [66,70,90]. Moreover, Zhang et al. [91] found that the interaction between the continuous accumulation of PM_2.5_ and further deterioration of meteorological conditions resulted in the explosive rise of PM_2.5_ in the middle period of HPEs.

It is worth noting that the hourly mean of PM_2.5_ concentrations during HPE6 peaked among six pollution episodes, increasing sharply from 110 μg m^−3^ on the evening of March 14 to exceeding 600 μg m^−3^ on the morning of March 15. The explosive increase in PM_2.5_ could be explained by the regional transport under favorable meteorology with high BLH and wind and low humidity. This differs from the previous studies that the two-way feedback between unfavorable meteorology and accumulated PM_2.5_ is the dominant mechanism for the occurrence of explosive increase [91].

**Figure 5:**
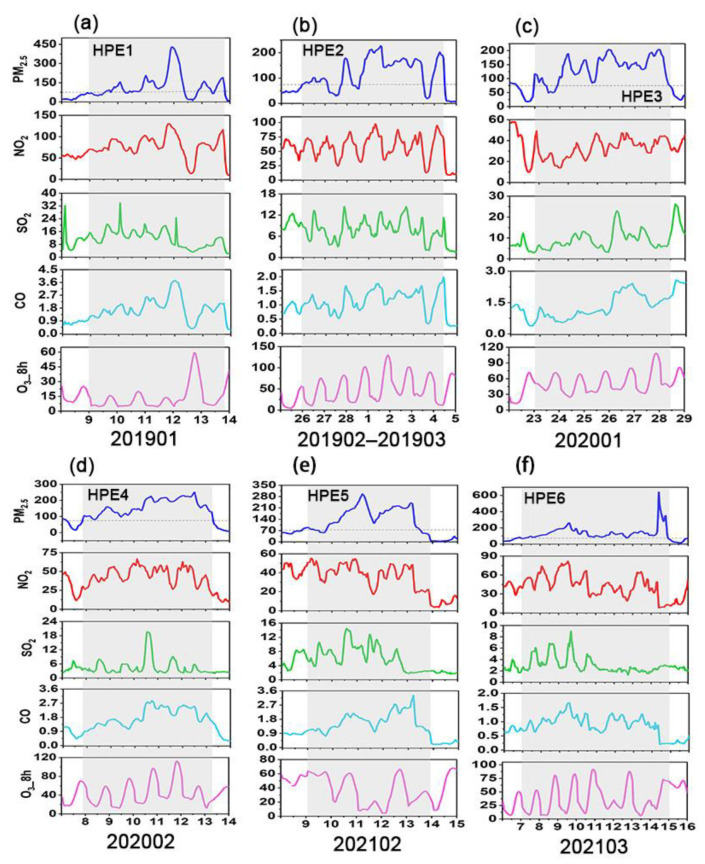
Time series of air pollutant concentrations during six heavy PM_2.5_ pollution episodes (HPEs) in Beijing. (**a**) HPE1, (**b**) HPE2, (**c**) HPE3, (**d**) HPE4, (**e**) HPE5, and (**f**) HPE6. PM_2.5_, NO_2_, SO_2_, and O_3__8h concentrations are in μg m^−3^, and CO concentrations are in mg m^−3^.

Furthermore, the variations in gaseous pollutants were investigated during the six HPEs (Figure 5). The gaseous pollutants (excluding O_3_) were generally characterized by remarkably elevated concentrations. It was observed that the SO_2_ and CO concentrations during the six HPEs increased by 6.44–56.55% and 32.89–114.32%, respectively, compared with the corresponding monthly average concentrations. For NO_2_, except for HPE3, the concentrations increased by 18.59–81.08% during the pollution episodes. In contrast, the NO_2_ concentration decreased by 16.38% during the HPE3, mainly due to the drastic reduction in traffic caused by the COVID-19 lockdown. Gao et al. [83] revealed that diffusive sources of NO_2_ were mainly related to vehicular traffic. As for O_3_, the concentrations decreased by 3.08–51.98% during the period of HPE1, HPE4, HPE 5, and HPE6, whereas the concentrations increased by 6.61% and 46.19%, respectively, in the HPE2 and HPE3. A significant increase in O_3_ concentration in the HPE3 was associated with the reduced NO_2_ that hindered the reaction between NO and O_3_, resulting in the increased atmospheric oxidizing capacity [92,93,94]. A similar phenomenon was also observed in an air pollution episode in Shijiazhuang during the COVID-19 outbreak [66]. Several studies also revealed that the drop in NO_x_ could not avoid PM_2.5_ pollution during the COVID-19 outbreak [18,95]. The variations in gaseous precursors indicated that the secondary formation was a critical process during pollution events.

The ratio of PM_2.5_/CO is an indicator of secondary pollutants to primary emissions [65]. Specifically, the ratio of PM_2.5_/CO during six HPEs increased by 46.96%, 68.09%, 88.37%, 53.22%, 35.13%, and 42.46%, respectively, compared with the corresponding monthly average PM_2.5_/CO ratio. These pronounced increases in PM_2.5_/CO ratio further revealed the great contribution of secondary formation to heavy PM_2.5_ pollution in Beijing. Consistently, studies have revealed that the persistent severe haze pollution in BTH and eastern China was associated with stronger secondary formation under a stagnant atmosphere despite the great reductions in primary emissions during the COVID-19 lockdown [65,96,97].

**Figure 6:**
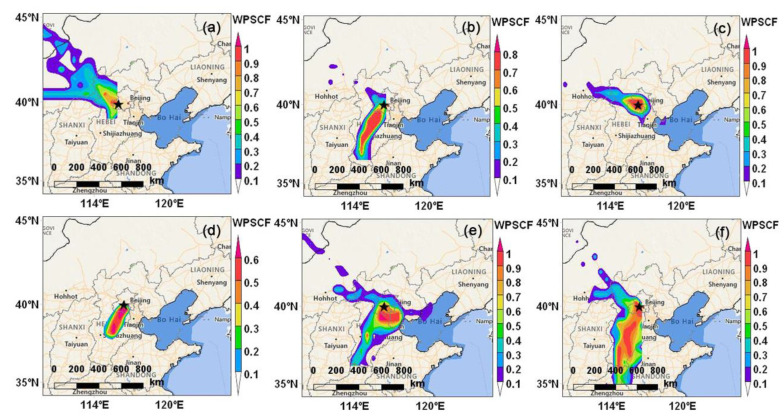
Maps of weighted potential source contribution function (WPSCF) for PM_2.5_ arriving in Beijing during the six heavy PM_2.5_ pollution episodes (HPEs). (**a**) HPE1, (**b**)HPE2, (**c**) HPE3, (**d**) HPE4, (**e**) HPE5, and (**f**) HPE6.

As illustrated in Figure 6, the potential source regions of PM_2.5_ during six PM_2.5_ pollution episodes were investigated by applying the WPSCF model. The relatively small areas with high WPSCF values were observed in HPE1, HPE3, and HPE5, indicating the crucial influence from surrounding areas, including western and southern Hebei, as well as Tianjin. In comparison, the wide banded areas with high WPSCF values were observed during the period of HPE2, HPE4, and HPE6, especially during HPE6, and the potential source regions were mainly in the southern Hebei, which indicated the industrial emissions contributed to this HPE because Hebei is the largest industrial cluster in China [98]. The potential source regions of PM_2.5_ for the HPE2, HPE4, and HPE6 revealed that long-distance regional transport played a significant role in causing PM_2.5_ pollution. Hence, more strengthened regional collaborative air pollution control in Beijing and the surrounding provinces is suggested. Overall, regional transport, secondary formation, along with adverse meteorology work jointly to the formation and evolution of heavy PM_2.5_ pollution in Beijing, which is a complex process.

#### 3.3.2. Analysis of PM_2.5_ Episodes on the Southern Edge of the NCP

**Figure 7:**
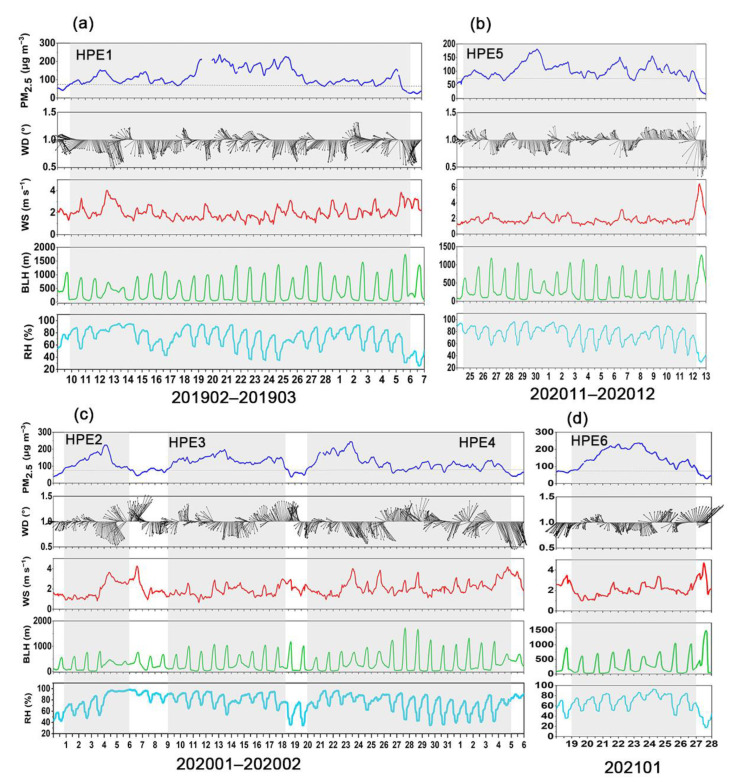
Time series of PM_2.5_ concentrations, wind direction (WD), wind speed (WS), boundary layer height (BLH), and relative humidity (RH) during six heavy PM_2.5_ pollution episodes (HPEs) in Henan. (**a**) HPE1, (**b**) HPE5, (**c**) HPE2, HPE3, and HPE4, and (**d**) HPE6.

For Henan, the formation mechanism of PM_2.5_ pollution between 2019 and 2021 was also explored with a detailed analysis of six HPEs (Table 3 and Figure 7). In contrast with Beijing, the duration of PM_2.5_ pollution in Henan was longer. Specifically, HPE1 lasted twenty-four days, including six consecutive days with PM_2.5_ levels reaching heavy pollution (over 150 μg m^−3^). In 2020, from January to early February, there were three persistent PM_2.5_ pollution events for 29 days. Moreover, from November to December of 2020, a seventeen-day PM_2.5_ pollution incident occurred. Except for HPE4, the average WS during pollution episodes was below 2 m s^−1^ with predominantly northerly winds, which led to the accumulation of air pollutants in Henan from the northern areas with high emission loads along the weak northerly winds. Furthermore, the average values of RH during six HPEs were 75%, and the BLH also decreased remarkably; thereby, PM_2.5_ accumulated in Henan under constant high humidity and stable atmospheric conditions, resulting in persistent PM_2.5_ pollution. In addition to unfavorable meteorological conditions, the socio-economic factors, such as economic development level, industrial structure, energy consumption, scientific level, and land-use structure contributed to the longer duration of PM_2.5_ pollution in Henan compared to Beijing [99,100], which was also indicated in Section 2.1. Jiang et al. [101] suggested that the central region, including Henan, should endeavor to finish industrial restructuring and remove the backward production capacity.

**Figure 8:**
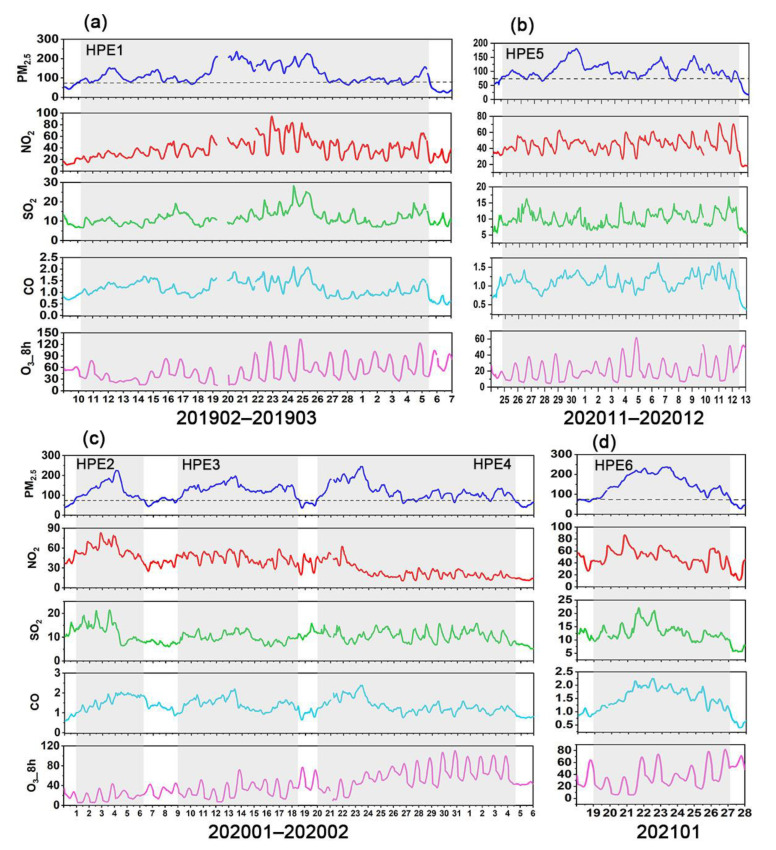
Time series of air pollutant concentrations during six heavy PM_2.5_ pollution episodes (HPEs) in Henan. (**a**) HPE1, (**b**) HPE5, (**c**) HPE2, HPE3, and HPE4, and (**d**) HPE6. PM_2.5_, NO_2_, SO_2_, and O_3__8h concentrations are in μg m^−3^, and CO concentrations are in mg m^−3^.

The variations in gaseous precursors are present in Figure 8. The CO concentrations during the six HPEs increased by 4.26–42.34% compared to the corresponding monthly average concentrations. The NO_2_ concentrations increased by 6.59–50.61%, but decreased by 18.51% during the HPE4, similar to Beijing. During the period of HPE3 in Beijing and HPE4 in Henan, the decrease in NO_2_ concentration was mainly attributed to the lockdown caused by COVID-19. As for SO_2_, the concentrations increased by 3.90–23.33% during the HPE2, HPE4, and HPE6, whereas the concentrations decreased by 1.88–8.15% during the HPE1, HPE3, and HPE5. Unlike other gaseous pollutants, O_3_ concentration decreased by 1.82–52.40%, but increased by 24.13% during the HPE4. An increased O_3_ concentration during the HPE4 in Henan was mainly attributed to the decreased NO_2_ concentration, consistent with what was discussed in Section 3.3.1.

Further, the ratio of PM_2.5_/CO during the six HPEs increased by 17.21%, 4.55%, 17.27%, 10.71%, 25.86%, and 35.44%, respectively, compared with the corresponding monthly ratio. Unlike Beijing, the impact of secondary formation on PM_2.5_ pollution in Henan was relatively small. This is consistent with the findings by Huang et al. [65], revealing the relatively higher secondary PM_2.5_ production in the BTH region. Some studies have revealed the dominant proportions of primary emission to the PM_2.5_ pollution in southern parts of the NCP compared to the secondary formation [73], which was consistent with our results.

**Figure 9:**
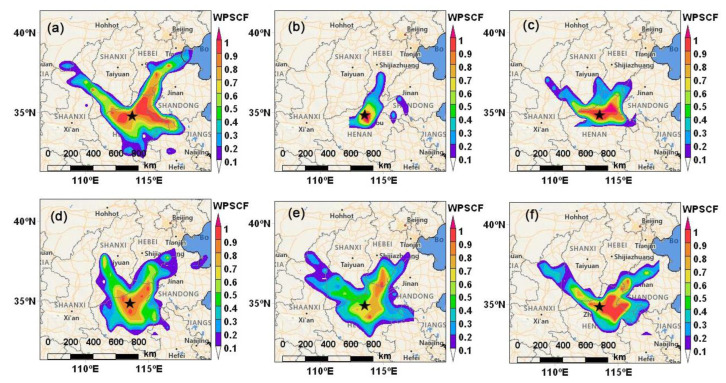
Maps of weighted potential source contribution function (WPSCF) for PM_2.5_ arriving in Henan during the six heavy PM_2.5_ pollution episodes (HPEs). (**a**) HPE1, (**b**)HPE2, (**c**) HPE3, (**d**) HPE4, (**e**) HPE5, and (**f**) HPE6.

The WPSCF map for the six PM_2.5_ pollution episodes in Henan showed that the potential source regions, except for Henan itself, were mainly in the areas adjacent to Henan, including southern Hebei, southern Shanxi, and western Shandong (Figure 9). This indicated that PM_2.5_ pollution sources were featured by local dispersion and small-scale regional transport rather than long-distance regional transport, and similar conclusions were also found by Song et al. [73]. Overall, PM_2.5_ pollution was dominated by local accumulation with a higher proportion of primary emissions under the persistent high humidity and stable atmospheric conditions, while superimposed regional transport on a small scale.

## 4. Conclusions

This study evaluated the change trends of PM_2.5_ and gaseous pollutants from 2017 to 2021 in Beijing and Henan and further explored the drivers for the PM_2.5_ pollution episodes pre- to post-COVID-19 (2019–2021) based on the air pollutant observation dataset. The main findings are as follows:

(1) The annual concentrations of PM_2.5_, NO_2_, SO_2_, and CO decreased year by year during 2017–2021 under a series of clean air action plans, whereas the exception was NO_2_ in Beijing in 2021, which increased slightly by 3.6% relative to 2020. During 2017–2021, the concentrations of PM_2.5_, SO_2_, and CO in Henan were higher than in Beijing. In contrast, NO_2_ concentration was the opposite, except in 2020. The differences in pollutant levels between Beijing and Henan are related to pollution emissions, development levels, and other socio-economic indicators. Unlike other gaseous pollutants, the 90th percentiles of MDA8 O_3_ values began to decrease significantly in 2020 in Beijing and Henan.
(2) The lockdown measures to constrain COVID-19 significantly improved air quality, and the concentrations of PM_2.5_, NO_2_, SO_2_, and CO decreased sharply in February 2020. The exceptions were PM_2.5_ and CO in Beijing, which exhibited a delayed decrease in March caused by adverse meteorological conditions and transported pollutants emitted by non-stop industries and fireworks and reached the lowest values relative to March of 2017–2019 and 2021.
(3) Overall, the PM_2.5_ pollution conditions have improved significantly. However, Beijing and Henan still suffered from heavy PM_2.5_ pollution between 2019 and 2021. For Beijing, the formation and evolution of PM_2.5_ pollution were caused by initial regional transport and following secondary formation under adverse meteorology. Unlike Beijing, PM_2.5_ elevation in Henan was caused by local accumulation with a dominated proportion of primary emissions under adverse atmospheric conditions, superimposing regional transport on a small scale. Hence, the heavy PM_2.5_ pollution on the NCP was highly heterogeneous, and stagnant weather, such as low wind speed, shallow boundary layer, and high humidity, is one of the major drivers of heavy PM_2.5_ pollution on the NCP.
(4) The formation and evolution of elevated PM_2.5_ pollution are affected by multiple factors. A balanced and coordinated strategy in regulating various air pollutants, the critical role of meteorology, and strengthened regional collaborative air pollution control should be considered when setting mitigation measures on the NCP.

Understanding the contributions of different sources to PM_2.5_ pollution and identifying the dominant emission sources are essential while formulating an effective air pollution strategy. However, the present study has some limitations. The contributions of sources to PM_2.5_ pollution were not examined in detail in the present study, especially for the period of heavy PM_2.5_ pollution. Therefore, subsequent studies shall perform source apportionments of PM_2.5_ to reveal the emission sources with more detailed information.

## Figures and Tables

**Table 1:** Proportion (%) of different concentration levels during 2017–2021 in Beijing and Henan.

PM_2.5_ Concentrations (μg m^−3^)	Beijing					Henan				
	2017	2018	2019	2020	2021	2017	2018	2019	2020	2021
≤35	43.1	42.2	50.3	57.8	69.4	15.4	25.0	39.3	43.1	49.2
35–75	33.1	35.8	36.2	32.5	19.2	58.3	49.9	34.4	37.0	33.7
75–115	14.3	13.6	10.0	6.1	5.8	13.4	14.0	15.1	11.9	13.3
115–150	3.9	4.2	2.3	1.9	3.9	6.7	4.5	3.7	5.8	1.9
150–250	4.5	4.2	1.2	1.7	1.7	5.9	6.3	7.5	2.2	1.9
>250	1.1	0.0	0.0	0.0	0.0	0.3	0.3	0.0	0.0	0.0

**Table 2:** Six heavy PM_2.5_ pollution episodes (HPEs) in Beijing pre- to post-COVID-19.

Event	Date	Duration (Day)	Mean PM_2.5_ Concentrations (μg m^−3^)	Maximum Hourly PM_2.5_ Concentration (μg m^−3^)
HPE1	10 January–14 January 2019	5	123.87	428.50
HPE2	27 February–5 March 2019	7	117.42	227.08
HPE3	24 January–28 January 2020	5	138.70	204.83
HPE4	9 February–13 February 2020	5	166.45	250.50
HPE5	10 February–14 February 2021	5	151.31	296.00
HPE6	8 March–15 March 2021	8	143.16	639.91

**Table 3:** Six heavy PM_2.5_ pollution episodes (HPEs) in Henan pre- to post-COVID-19.

Event	Date	Duration (Day)	Mean PM_2.5_ Concentrations (μg m^−3^)	Maximum Hourly PM_2.5_ Concentration (μg m^−3^)
HPE1	11 February–6 March 2019	24	119.35	236.97
HPE2	2 January–6 January 2020	5	135.88	224.65
HPE3	10 January–18 January 2020	9	138.60	197.83
HPE4	21 January–5 February 2020	15	115.90	244.73
HPE5	26 November–12 December 2020	17	105.93	180.67
HPE6	20 January–27 January 2021	8	160.85	236.96

## Data Availability

The data on PM_2.5_ and gaseous pollutants are available at https://quotsoft.net/air/#archive (accessed on 27 July 2022). The meteorology data is available at http://cds.climate.copernicus.eu/cdsapp#/home (accessed on 27 July 2022), and meteorological inputs to the backward trajectories are available at ftp://arlftp.arlhq.noaa.gov/pub/archives/gdas1 (accessed on 27 July 2022).

## Supplementary Materials

The following supporting information can be downloaded at: https://www.mdpi.com/article/10.3390/ijerph191912904/s1, Table S1: Locations of the ground-level monitoring sites; Table S2: Statistical description of PM_2.5_ and gaseous pollutants during 2017–2021 in Beijing and Henan.
