# Supplementary material for: Changes in Air Quality and Drivers for the Heavy PM2.5 Pollution on the North China Plain Pre- to Post-COVID-19
Source: Int J Environ Res Public Health. 2022 Oct 8;19(19):12904. doi: 10.3390/ijerph191912904 (PMC9566441; doi:10.3390/ijerph191912904)
Supplement: Supplementary file 1 [file ijerph-19-12904-s001.zip › ijerph-1918332-supplementary.pdf]

Changes in Air Quality and Drivers for the Heavy $PM_{2.5}$ Pollution on the North China Plain Pre- to Post-COVID-19

Shuang Liu, Xingchuan Yang, Fuzhou Duan *, and Wenji Zhao *

College of Resource Environment and Tourism, Capital Normal University, Beijing 100048, China
* Correspondence: 5482@cnu.edu.cn (F.D.); 4973@cnu.edu.cn (W.Z.)

Table S1. Locations of the ground-level monitoring sites.

No.	Station code	Longitude	Latitude
1	1001A	116.366	39.8673
2	1002A	116.17	40.2865
3	1003A	116.434	39.9522
4	1004A	116.434	39.8745
5	1005A	116.473	39.9716
6	1006A	116.361	39.9425
7	1007A	116.315	39.9934
8	1008A	116.72	40.1438
9	1009A	116.644	40.3937
10	1010A	116.23	40.1952
11	1011A	116.407	40.0031
12	1012A	116.225	39.9279
13	1316A	113.681	34.7545
14	1317A	113.641	34.7745
15	1318A	113.675	34.8019
16	1319A	113.564	34.802
17	1320A	113.727	34.7187
18	1321A	113.734	34.719
19	1322A	113.5991	34.7496
20	1323A	113.6356	34.7538
21	1324A	113.6113	34.9162
22	1811A	112.3628	34.6692
23	1812A	112.3939	34.6511
24	1813A	112.4333	34.67
25	1814A	112.4664	34.6869
26	1815A	112.4831	34.6869
27	1816A	112.3844	34.6231
28	1817A	112.4275	34.6258
29	1818A	114.483	36.061
30	1819A	114.32	36.086
31	1820A	114.358	36.087

32	1821A	114.3931	36.0883
33	1822A	114.286	36.11
34	1823A	114.3406	34.8022
35	1824A	114.3389	34.7781
36	1825A	114.3733	34.7975
37	1826A	114.2886	34.7967
38	1827A	113.261	35.235
39	1828A	113.227	35.213
40	1829A	113.2464	35.1764
41	1830A	113.1992	35.259
42	1831A	113.3064	33.7214
43	1832A	113.322	33.721
44	1833A	113.292	33.739
45	1834A	113.182	33.737
46	1835A	111.1928	34.7772
47	1836A	111.158	34.794
48	1837A	111.1858	34.7917
49	1838A	111.1489	34.7997
50	2054A	114.1044	32.1078
51	2064A	114.0122	32.1403
52	2065A	114.0681	32.1342
53	2066A	114.0614	32.1383
54	2067A	114.6613	33.6128
55	2068A	114.6758	33.6347
56	2069A	114.6546	33.5979
57	2070A	114.6369	33.6406
58	2385A	114.17	35.9019
59	2386A	114.2956	35.7511
60	2387A	114.2878	35.7306
61	2388A	113.926	35.279
62	2389A	113.836	35.31
63	2390A	113.884	35.272
64	2391A	113.883	35.303
65	2392A	115.031	35.763
66	2393A	114.9906	35.767
67	2394A	115.0628	35.7672
68	2395A	115.0772	35.767
69	2396A	113.8331	34.0117
70	2397A	113.8208	34.0314
71	2398A	113.7906	33.9953
72	2399A	114.056	33.567
73	2400A	114.005	33.568
74	2401A	114.014	33.581
75	2402A	114.0322	33.5653

76	2403A	112.5192	32.9917
77	2404A	112.5224	33.0122
78	2405A	112.5003	32.9735
79	2406A	112.5573	33.027
80	2407A	112.55	32.9683
81	2408A	115.6697	34.4286
82	2409A	115.6558	34.429
83	2410A	115.6386	34.407
84	2411A	115.6578	34.402
85	2420A	114.018	32.965
86	2421A	114.0131	33.0069
87	2422A	113.996	32.996
88	3021A	115.0061	35.76806
89	3022A	112.4433	34.6686
90	3039A	111.19	34.7822
91	3054A	113.5922	35.28385
92	3134A	113.8172	34.0339
93	3141A	114.3922	36.0878
94	3147A	114.3442	34.8097
95	3169A	113.2725	35.2067

No. 1–No. 12 are in Beijing, and the rest of the sites are in Henan.

Table S2. Statistical description of $PM_{2.5}$ and gaseous pollutants during 2017–2021 in Beijing and Henan.

Pollutants	Statistics	Beijing					Henan				
		2017	2018	2019	2020	2021	2017	2018	2019	2020	2021
$PM_{2.5}$	Mean	56	51	42	38	35	67	63	60	52	47
(μg m^{-3})	SD	51	43	32	32	37	42	42	44	36	33
	Median	40	42	35	29	23	52	49	42	38	36
	Min	6	5	4	4	3	16	13	11	12	7
	Max	344	243	200	208	235	257	262	232	207	218
	Change (%)		−8.9	−17.6	−9.5	−7.9		−6.0	−4.8	−13.3	−9.6
NO_2	Mean	44	40	36	28	29	41	37	35	30	27
(μg m^{-3})	SD	20	19	17	15	14	15	14	14	13	13
	Median	39	36	32	24	25	37	35	32	28	24
	Min	11	7	8	4	4	17	16	12	10	8
	Max	146	102	97	94	83	84	84	76	70	70
	Change (%)		−9.1	−10.0	−22.2	3.6		−9.8	−5.4	−14.3	−10.0
SO_2	Mean	7	6	4	4	3	21	14	11	9	8
(μg m^{-3})	SD	7	4	3	2	1	10	5	4	3	3
	Median	5	5	3	3	3	19	14	10	9	8
	Min	2	2	2	2	2	6	6	4	4	4
	Max	44	25	21	13	9	54	38	26	17	21
	Change (%)		−14.3	−33.3	0.0	−25.0		−33.3	−21.4	−18.2	−11.1
CO	Mean	0.9	0.8	0.7	0.6	0.6	1.3	1.2	0.9	0.8	0.7

(mg m^{-3})	SD	0.7	0.4	0.4	0.4	0.3	0.5	0.3	0.3	0.2	0.2
	Median	0.8	0.8	0.6	0.6	0.6	1.2	1.0	0.8	0.7	0.7
	Min	0.2	0.2	0.2	0.1	0.2	0.7	0.6	0.5	0.4	0.4
	Max	7.3	2.6	2.5	2.3	2.3	4.6	2.8	2.1	2.1	2.0
	Change (%)		−11.1	−12.5	−14.3	0.0		−7.7	−25.0	−11.1	−12.5
MDA8 O_3	90th percentile	195	198	196	176	158	182	178	182	168	161
(μg m^{-3})	Change (%)		1.5	−1.0	−10.2	−10.2		−2.2	2.2	−7.7	−4.2
